# Geographic Distribution of *Ehrlichia canis* TRP Genotypes in Brazil

**DOI:** 10.3390/vetsci7040165

**Published:** 2020-10-29

**Authors:** Isis Indaiara Gonçalves Granjeiro Taques, Amanda Noeli Silva Campos, Mayara Lima Kavasaki, Sayanne Luns Hatum de Almeida, Daniel Moura de Aguiar

**Affiliations:** 1 Laboratory of Virology and Rickettsial Infections, Veterinary Hospital, Federal University of Mato Grosso, Av. Fernando Correa da Costa 2367, Cuiabá 78090-900, Brazil; isis_indaiara@hotmail.com (I.I.G.G.T.); amanda.noeli@hotmail.com (A.N.S.C.); mayarakawa@gmail.com (M.L.K.); 2 Laboratory of Parasitology and Parasitic Diseases, Veterinary Hospital, Federal University of Mato Grosso, Av. Fernando Correa da Costa 2367, Cuiabá 78090-900, Brazil; sayhatum@gmail.com

**Keywords:** dogs, ehrlichiosis, ELISA, tick-borne, TRP36, TRP19

## Abstract

Tandem repeat proteins (TRPs) are major immunoreactive proteins of *Ehrlichia canis*, which have been used in the serological diagnosis of different genotypes of the microorganism. TRP19 is preserved among different *E. canis* isolates expressed on both reticulate and dense-core cells and observed in the extracellular matrix or associated with the morula membrane. TRP36 is differentially expressed only on the surface of the dense-core form of the bacterium and exhibits more divergence among isolates. The aim of this study was to evaluate the distribution of the American (USTRP36), Brazilian (BrTRP36) and Costa Rican (CRTRP36) genotypes of *E. canis* in Brazil, using ELISA assays. Serum samples of 814 dogs from 49 municipalities from all over Brazil were analyzed. Our results showed that 34% of the samples were reactive to the USTRP36 genotype and 32.6% to the BrTRP36 genotype. The two genotypes appeared to occur equally throughout Brazil, although the frequency of seropositivity was lower in the south than in the country’s other regions. Dogs that reacted to at least one of the synthetic peptides (TRP19 and TRP36) were 456 (56%). A few dogs (*n* = 5; 0.61%) reactive to the *E. canis* TRP36 genotype (CRTRP36) were also detected in the northeast and southern regions. We concluded that the American and Brazilian genotypes of *E. canis* are distributed evenly in Brazil, especially in the tropical region, while the temperate region in the south presented the lowest prevalence rates. This study offers the first report of dogs seropositive for the Costa Rican genotype in Brazil.

## 1. Introduction

*Ehrlichia canis* is the etiologic agent of canine monocytic ehrlichiosis (CME), a serious tick-borne disease of worldwide distribution [1]. A group of tandem repeat proteins of 19 and 36 kDa (TRP19 and TRP36) were characterized as important immunoreactive proteins of *E. canis* and were employed in the serological diagnosis of CME [2,3].

TRP19 is highly conserved among the known *E. canis* strains and was found on both reticulate and dense-core cells. Although they appeared to be located predominantly in the cytoplasm of Ehrlichia, they also appeared to be present outside the morula, suggesting that they may be involved in host cell interactions during infection. TRP19 is a specific target for serological diagnostics [3,4,5,6] and a strong candidate for vaccine antigen against CME, due to its high degree of conservation and because it is a component of the morula membrane [7].

TRP36 provides more information about the genetic diversity of *E. canis* due to the divergence within amino acid tandem repeat sequences and/or in the number of tandem repeats [6,8,9,10]. The TRP36 protein is secreted by *E. canis* at the time of intracellular colonization and because it is involved in the adaptive process between the microorganism and the host, it has vaccine potential [3]. *Ehrlichia canis* TRP36 synthetic peptide antigens were used in enzyme-linked immunosorbent assays (ELISA) by Aguiar et al. [4], who found dogs seroreactive to two distinct genotypes in Brazil: American (USTRP36) and Brazilian (BrTRP36). Recently, dogs seropositive to the new zoonotic *E. canis* TRP36 genotype described in Costa Rica (CRTRP36) were identified in Colombia [9,11].

In view of the epidemiological importance of CME in Brazil, and the antigenic relevance of these proteins, canine blood samples were obtained from various regions of Brazil to be evaluated in serological assays using crude *E. canis* antigens by immunofluorescence assay (IFA) and synthetic peptides from TRP19 and 36 (American, Brazilian and Costa Rican genotypes) by ELISA. Moreover, the use of TRP36 antigens revealed the wide distribution of different genotypes throughout Brazil and the first report of dogs seropositive for the Costa Rican genotype in Brazil.

## 2. Materials and Methods

This study included serum samples from 814 dogs suspected of CME in 49 municipalities (Table 1 and Table 2) all over Brazil and treated at veterinary clinics and hospitals and zoonosis control centers. Canine serum samples from the city of Palotina, in the state of Paraná (*n* = 30) were previously selected and analyzed, since one of them was known to be *E. canis* positive (SNAP 4Dx**^®^** Plus Test IDEXX Laboratories©, Westbrook, Maine). The presence of anti-*Ehrlichia* spp. serum antibodies was determined by IFA, using crude antigens of the Cuiaba#1 strain of *E. canis* [12]. ELISA was performed with synthetic peptides corresponding to epitopes from *E. canis* TRP19 (HFTGPTSFEVNLSEEEKMELQEVS) [7], USTRP36 (TEDSVSAPATEDSVSAPA) [3], BrTRP36 (ASVVPEAEASVVPEAEASVVPEAE) [4] and CRTRP36 (EASVVPAAEAPQPAQQTEDEFFSDGIEA) [11]. All the ELISA assays were performed according to a previously described protocol [4,5]. Differences between the results found per region and *E. canis* TRP36 peptides were evaluated by the chi-square test and *p* ≤ 0.05 was considered significant. The statistical analysis was performed using Epi Info™ version 5.5.1 software. This study was approved by the Committee on Animal Research and Ethics of the Federal University of Mato Grosso (UFMT) under protocol no. 23108.122592/2015-10.

## 3. Results

**Figure 1:**
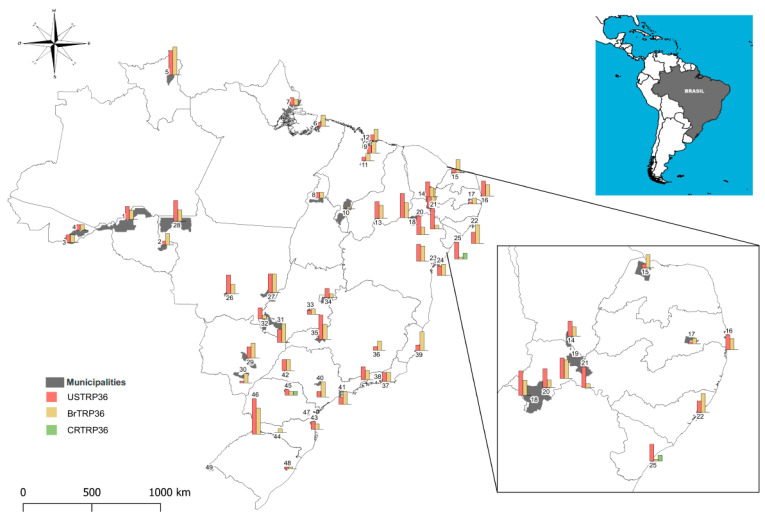
Distribution of *E. canis* genotypes in Brazil.

The average number of samples evaluated per city was 14.5 in the center west, 13 in the northeast, 15.25 in the north, 15.3 in the southeast and 28.8 in the south. Table 1 and Table 2 list the number of dogs tested and seropositive in serological assays per city and region. A total of 349 (42.8%) dogs had antibodies to the TRP19 peptide, 277 (34%) had antibodies to the USTRP36 peptide, 266 (32.6%) had antibodies to the BrTRP36 peptide and 5 (0.61%) dogs had antibodies to the CRTRP36 peptide. Dogs that reacted to at least one of the synthetic peptides (TRP19 and TRP36) were 456 (56%). Dual reactivity in dogs positive for USTRP36 and BrTRP36 peptides was detected in 131 samples (16.0%) from all the regions. The frequency of seropositivity for any of the TRP36 peptides was 50.7% (413/814). Reactive solely to TRP19 were 40 (4.9%) and to TRP36 were 104 (12.7%). Three hundred and seventy-five (46%) dogs were seroreactive to IFA. Overall, similar results were observed among the dogs reactive to USTRP36 and BrTRP36 in all the Brazilian regions (*p* > 0.05) (uppercase letters in Table 2). However, the values showed differences when evaluated as a function of antigen (lowercase letters in Table 2). The southern region, for example, differed from the others in all the tests (*p* < 0.05). Exceptionally, samples from Barra do Quaraí and Curitiba were negative for IFA, and all the peptides and reactions against CR TRP36 peptides were observed only in Aracaju, SE and Londrina, PR. Figure 1 shows the location of each city and the ELISA results using TRP36 synthetic peptides.

## 4. Discussion

The present study involved a serological survey in dogs from all over Brazil, based on the detection of antibodies against antigens of *E. canis* TRPs. Taking into account dogs that reacted against any of *E. canis* TRP synthetic peptides, the frequency observed was 56%. This value exceeded the result observed in the *E. canis*-IFA, which was 46%. A previous study provided a higher level of sensitivity when recombinant TRP19/36 *E. canis* proteins in an ELISA format was used compared to *E. canis*-IFA during the early stages of canine ehrlichiosis [2]. Dogs that reacted to at least one of the TRP36 peptides had a higher frequency of positivity than those that were positive for TRP19. This result was expected because experimentally infected dogs showed earlier reactivity to TRP36 than TRP19 [2].

In Brazil, with the exception of the southern states of Santa Catarina and Rio Grande do Sul [13], *Ehrlichia canis* infections in dogs are widespread around the country, showing high prevalence rates and high IFA titers [14]. Our study is the first to assess the distribution of *E. canis* genotypes based on the detection of specific antibodies against *E. canis* TRP36 synthetic peptides in dogs suspected of CME from all over Brazil. Our findings indicated that the American and Brazilian genotypes are widely distributed throughout the country’s various regions (Figure 1).

The presence of co-reaction between US and Br genotypes, suggestive of co-infection by the different TRP36 genotypes, was identified in all the regions. Co-infection by different genotypes in the same host may lead to the development of the genetic recombination of *E. canis*. This mechanism is commonly used by microorganisms as an immune evasion strategy and is also considered a major driver of genetic diversity in obligate intracellular microorganisms [15]. Examples of this phenomenon are reported in the isolate Cuiabá #16 of *E. canis* and several isolates of *E. ruminantium* [8,16]. As seen in other tick-borne pathogens [15], the genetic recombination process can generate new strains of *E. canis* with different degrees of virulence, which may be fatal to dogs. Furthermore, new strains may be able to infect and adapt to new hosts, as probably occurred with *E. canis* and *E. minasensis* in bovines [17].

Unfortunately, our study was limited to testing dogs suspected of CME. Complete clinical data of the dogs in this study were not available for reasons of medical confidentiality. No clinical differences between US or Br genotypes were observed in a hospital population of dogs suffering from CME in an endemic area in Brazil [5], while clinical differences were observed in dogs infected by different genotypes in another study [18]. To date, the genetic diversity may be related to the host–pathogen relationship. In this regard, a larger number of clinical cases of CME should be investigated in order to ensure more reliable conclusions about the clinical diseases and infections by different genotypes of *E. canis* in dogs.

Indeed, the fairly large number of co-infected dogs detected in Brazil supports the possibility of the emergence of new genotypes of *E. canis*. In our study, 40 (4.9%) dogs reactive only to TRP19 peptide suggests that these dogs may have been exposed to a to date undescribed *E. canis* TRP36 genotype circulating in Brazil.

Despite the low detection of antibodies against the Costa Rica genotype in our study (*n* = 5; 0.61%), this genotype may also have been a target of genetic recombination, since its amino acid sequence is similar to that of BrTRP36 [9,18]. In Brazil, this genotype was found pointwise in Aracajú—northeast region (three dogs) and Londrina—south region (two dogs). The genotype CRTRP36 was first detected in the blood of human donors in Costa Rica, exhibiting a potential zoonotic relationship [9]. However, data from South American serosurveys reveal evidence of infection by this genotype only in dogs from Colombia and Peru [11,19].

Our findings confirmed the low occurrence of seroreactive dogs in the south of Brazil. Similar data have been reported but are based only on the IFA test [13]. In the present study, with the exception of samples from the city of Palotina, PR, the other samples showed IFA results (~7.5%; 13/172 samples) similar to those reported [13]. However, 15% (2/13) of these samples presented seronegative results in all the ELISA assays using synthetic peptides of *E. canis*. This suggests that some of the reactions observed in dogs by IFA in the south are nonspecific, probably reflecting cross-reactions produced during antigenic stimulation after infections by closely related agents such as *A. platys* or unknown *Ehrlichia* species [20]. It should be noted that a relevant factor influencing the low prevalence of *E. canis* infection in the south of Brazil is the presence of the temperate strain of *R. sanguineus* ticks established in the region, which has low vector competence for the agent [21].

## 5. Conclusions

We conclude, from this study, that *E. canis* genotypes based on the TRP36 protein are distributed equally throughout Brazil. Comparisons between IFA and ELISA TRP19 reveal the occurrence of false positive reactions resulting from cross-reactions with other agents similar to *E. canis* in the indirect immunofluorescence reaction. Brazil’s southern region has lower rates of seropositive dogs than other Brazilian regions. This paper reports for the first time dogs seropositive for the Costa Rican genotype of *E. canis* in Brazil.

## Figures and Tables

**Table 1:** Number of sampled and positive dogs in serological assays by city and region in Brazil.

Location *	Map Reference	No. of Dogs	ELISA (%)				IFA (Immunofluorescence Assay) (%)
			TRP19	USTRP36	BrTRP36	CRTRP36	
North		122	65 (53.2)	38 (31.1)	45 (36.8)	0	58 (47.5)
Porto Velho, RO	1	15	11	7	5	0	9
Cacoal, RO	2	15	8	2	6	0	8
Brasiléia, AC	3	13	8	4	4	0	6
Rio Branco, AC	4	16	9	3	3	0	10
Boa Vista, RR	5	18	14	13	15	0	12
Belém, PA	6	16	5	2	6	0	4
Macapá, AP	7	15	4	4	3	0	3
Araguaína, TO	8	14	6	3	3	0	6
Northeast		236	114 (48.3)	111 (47.0)	94 (39.8)	3 (1.27)	124 (52.5)
São Luís, MA	9	18	7	4	6	0	4
Balsas, MA	10	9	1	0	1	0	1
Bacabal, MA	11	10	4	2	4	0	4
Raposa, MA	12	12	5	3	6	0	7
Guaribas, PI	13	14	8	9	7	0	9
Crato, CE	14	15	9	8	5	0	10
Mossoró, RN	15	14	7	2	7	0	8
João Pessoa, PB	16	14	5	8	6	0	7
Campina Grande, PB	17	10	4	1	3	0	3
Petrolina, PE	18	15	11	13	8	0	15
Serrita, PE	19	15	6	11	10	0	11
Lagoa Grande, PE	20	15	8	10	4	0	8
Salgueiro, PE	21	20	3	11	2	0	2
Maceió, AL	22	15	13	6	10	0	10
Cruz das Almas, BA	23	10	6	9	8	0	9
Salvador, BA	24	15	9	5	6	0	8
Aracaju, SE	25	15	8	9	1	3	8
Central West		131	75 (57.2)	58 (44.2)	51 (38.9)	0	87 (66.4)
Cuiabá, MT	26	15	15	10	5	0	15
Barra do Garças, MT	27	15	13	10	10	0	13
Colniza, MT	28	15	11	11	6	0	11
Campo Grande, MS	29	15	9	6	8	0	9
Dourados, MS	30	15	7	1	5	0	12
Jataí, GO	31	15	8	7	10	0	12
Mineiros, GO	32	15	4	6	2	0	7
Goiânia GO	33	11	5	2	3	0	5
Brasília, DF	34	15	3	5	2	0	3
Southeast		123	62 (50.4)	43 (34.9)	54 (43.9)	0	66 (53.6)
Uberlândia, MG	35	17	11	13	8	0	13
Itabirito, MG	36	16	4	2	5	0	4
Niterói, RJ	37	15	6	5	5	0	6
Seropédica, RJ	38	15	9	7	5	0	8
Vitória, ES	39	19	7	3	10	0	9
Botucatu, SP	40	15	12	3	8	0	15
São Paulo, SP	41	15	7	4	7	0	4
Pres. Prudente, SP	42	11	6	6	6	0	7
South		202	33 (16.3)	27 (13.3)	22 (10.8)	2 (0.9)	40 (19.8)
Joinville, SC	43	30	1	4	3	0	4
Concórdia, SC	44	40	2	0	2	0	1
Londrina, PR	45	14	5	3	2	2	6
Palotina, PR	46	30	25	19	14	0	27
Curitiba, PR	47	28	0	0	0	0	0
Porto Alegre, RS	48	30	0	1	1	0	2
Barra do Quaraí, RS	49	30	0	0	0	0	0
Total		814	349 (42.8)	277 (34)	266 (32.6)	5 (0.61)	375 (46)

* AC—Acre, AL—Alagoas, AP—Amapá, BA—Bahia, CE—Ceará, DF—Distrito Federal, ES—Espirito Santo, GO—Goiás, MA—Maranhão, MG—Minas Gerais, MS—Mato Grosso do Sul, MT—Mato Grosso, PA—Pará, PB—Paraíba, PE—Pernambuco, PR—Paraná, RJ—Rio de Janeiro, RN—Rio Grande do Norte, RO—Rondônia, RR—Roraima, RS—Rio Grande do Sul, SC—Santa Catarina, SE—Sergipe, SP—São Paulo, TO—Tocantins.

**Table 2:** Number and frequency of seropositive dogs according to serological assays, average and range of optical density by Tandem repeat proteins (TRPs) ELISA and titer range in IFA.

Region	No. of Dogs	Number of Positive Dogs (Frequency) (Optical Density Average; Range)				IFA (Frequency) (Titer Ranging)
		TRP19	USTRP36 *	BrTRP36 *	CRTRP36	
North	122	65 (53.2%) a (2.25; 0.42–3.77)	38 (31.1%) A b (1.21; 0.33–3.58)	45 (36.8%) A a (1.8; 0.33–3.8)	0	58 (47.5%) c b (40 to 10,240)
Northeast	236	114 (48.3%) a (2.05; 0.39–3.80)	111 (47.0%) A a (1.48; 0.33–3.47)	94 (39.8%) A a (2.06; 0.33–4.02)	3 (1.27%) (1.6; 1.04–2.13)	124 (52.5%) b (40 to 10,240)
Center West	131	75 (57.2%) a (2.22; 0.34–3.9)	58 (44.2%) A a (1.63; 0.33–3.24)	51 (38.9%) A a (1.82; 0.33–4.02)	0 (0%)	87 (66.4%) a (40 to 10,240)
Southeast	123	62 (50.4%) a (2.01; 0.35–3.74)	43 (34.9%) A b (1.45; 0.37–3.3)	54 (43.9%) A a (1.72; 0.36–3.96)	0	66 (53.6%) b (40 to 10,240)
South	202	33 (16.3%) b (2.33; 0.37–3.95)	27 (13.3%) A c (0.78; 0.33–2.51)	22 (10.8%) A b (1.6; 0.33–3.65)	2 (0.9%) (0.5; 0.35–0.66)	40 (19.8%) d (40 to 10,240)
TOTAL	814	349 (42.8%) (2.17; 0.34–3.95)	277 (34%) A (1.51; 0.33–3.58)	266 (32.6%) A (1.8; 0.33–4.02)	5 (0.61%) (1.05; 0.35–2.13)	375 (46%) (40 to 10,240)

* Same uppercase letter (A) on the same line indicates significantly similar values (*p* > 0.05). Different lowercase letters (a, b, c, d) in a column indicates significantly different values (*p* < 0.05).
